# Impact of targeted interventions on heterosexual transmission of HIV in India

**DOI:** 10.1186/1471-2458-11-549

**Published:** 2011-07-11

**Authors:** Rajesh Kumar, Sanjay M Mehendale, Samiran Panda, S Venkatesh, PVM Lakshmi, Manmeet Kaur, Shankar Prinja, Tarundeep Singh, Navkiran K Virdi, Pankaj Bahuguna, Arun K Sharma, Samiksha Singh, Sheela V Godbole, Arun Risbud, Boymkesh Manna, V Thirumugal, Tarun Roy, Ruchi Sogarwal, Nilesh D Pawar

**Affiliations:** 1 School of Public Health, Post Graduate Institute of Medical Education and Research, Sector 12, Chandigarh 160012, India; 2 National Institute of Epidemiology, R-127, 3rd Avenue, Tamil Nadu Housing Board, Ayapakkam, Chennai 600077, India; 3 National Institute of Cholera and Enteric Diseases. P-33, CIT Road, Scheme XM, Beleghata, Kolkata 700010, India; 4 National AIDS Control Organization. 9th Floor, Chandralok Building, 36, Jan path, New Delhi 110001, India; 5 Department of Community Medicine, University College of Medical Sciences, Dilshad Garden, New Delhi 110095, India; 6 National AIDS Research Institute. 73, 'G'-Block MIDC. Bhosari, Pune 411026, Maharashtra, India

**Keywords:** HIV, Impact, Evaluation, Condoms, Targeted Interventions, India

## Abstract

**Background:**

Targeted interventions (TIs) have been a major strategy for HIV prevention in India. We evaluated the impact of TIs on HIV prevalence in high HIV prevalence southern states (Tamil Nadu, Karnataka, Andhra Pradesh and Maharashtra).

**Methods:**

A quasi-experimental approach was used to retrospectively compare changes in HIV prevalence according to the intensity of targeted intervention implementation. Condom gap (number of condoms required minus condoms supplied by TIs) was used as an indicator of TI intensity. Annual average number of commercial sex acts per female sex worker (FSW) reported in Behavioral Surveillance Survey was multiplied by the estimated number of FSWs in each district to calculate annual requirement of condoms in the district. Data of condoms supplied by TIs from 1995 to 2008 was obtained from program records. Districts in each state were ranked into quartiles based on the TI intensity. Primary data of HIV Sentinel Surveillance was analyzed to calculate HIV prevalence reductions in each successive year taking 2001 as reference year according to the quartiles of TI intensity districts using generalized linear model with logit link and binomial distribution after adjusting for age, education, and place of residence (urban or rural).

**Results:**

In the high HIV prevalence southern states, the number of TI projects for FSWs increased from 5 to 310 between 1995 and 2008. In high TI intensity quartile districts (n = 30), 186 condoms per FSW/year were distributed through TIs as compared to 45 condoms/FSW/year in the low TI intensity districts (n = 29). Behavioral surveillance indicated significant rise in condom use from 2001 to 2009. Among FSWs consistent condom use with last paying clients increased from 58.6% to 83.7% (p < 0.001), and among men of reproductive age, the condom use during sex with non-regular partner increased from 51.7% to 68.6% (p < 0.001). A significant decline in HIV and syphilis prevalence has occurred in high prevalence southern states among FSWs and young antenatal women. Among young (15-24 years) antenatal clinic attendees significant decline was observed in HIV prevalence from 2001 to 2008 (OR = 0.42, 95% CI 0.28-0.62) in high TI intensity districts whereas in low TI intensity districts the change was not significant (OR = 1.01, 95% CI 0.67-1.5).

**Conclusion:**

Targeted interventions are associated with HIV prevalence decline.

## Background

India, the second most populous country of the world, has a highly heterogeneous HIV epidemic. Majority of the HIV infections are heterosexually transmitted, with unprotected paid sex being the major route of transmission. Besides being heterogeneous, the epidemic is concentrated, i.e., having high prevalence in the high risk groups (HRGs): injection drug users (IDU) (7.2%), men who have sex with men (MSM) (7.4%), female sex workers (FSW) (5.1%) and people with sexually transmitted infections (STI) (3.6%) [1]. The epidemic seems to be stabilizing as a decline in HIV has been observed in high HIV prevalence states of southern India [2,3].

National AIDS Control Program (NACP), initiated in India in 1992 by the National AIDS Control Organisation (NACO), is now in its third phase (2007-2012). It focuses on prevention of new infections by achieving saturation coverage (> 80%) of high risk groups (HRGs) with targeted interventions (TIs). These interventions comprise of safe behavior promotion (increased condom use and decreased needle sharing), and treatment of sexually transmitted infections (STIs).

Targeted intervention strategy is based on the premise that prevention of HIV transmission from FSWs to their male clients will result in lower rates of HIV transmission in their sexual contacts, i.e., women in general population. This should lead to lower HIV prevalence among the antenatal women, particularly those in the younger age groups, who are more likely to have become sexually active recently. Prevalence of HIV infection in young antenatal women has been considered as a surrogate for the incidence [4].

Small scale studies have indeed indicated that targeted HIV prevention interventions may help to stabilize the rates of HIV infection among sex workers [5,6], however, the contribution of TI strategy in stabilizing HIV epidemic in program settings have not been assessed. A wide range of interventions have been implemented in India with investment of 11,585 crore Rupees (USD 2.403 billion) in the third phase of NACP; of which 84% is to be spent for prevention, care and treatment [1]. The magnitude of resources invested in HIV prevention and control interventions warrant well designed impact evaluations. Hence, present study was conducted to assess the impact of TI strategy on HIV prevalence.

## Methods

Logical framework proposed by Parkhurst was used to evaluate the impact of program efforts on specific changes in the behaviors, sexually transmitted infections and HIV [7].

### Study design

Retrospective quasi-experimental analyses were carried out to compare syphilis and HIV trends among female sex workers and young pregnant women in districts having targeted interventions of different intensity.

### Study setting

Four southern states of India (Andhra Pradesh, Maharashtra, Karnataka and Tamil Nadu), which were more suited to test the study hypothesis, were chosen as (a) targeted interventions had been initiated in these states quite early in 1990s, (b) the prevalence of HIV was higher and had shown a declining trend [2] (c) HIV transmission was predominantly through sexual route (North-East States had more IDU).

### Data sources

Service delivery data of all Targeted Intervention (TI) Projects implemented between 1995 and 2008 were collected from the annual and semi-annual reports that were submitted by the TIs to respective State AIDS Control Societies (SACS) or their Development Partners. This data was also used to validate the information already available at the state and national levels through computerized management information system (CMIS).

Behavioral Surveillance Surveys (BSS) conducted in 2001, 2006 and 2009, and primary dataset of HIV Sentinel Surveillance (HSS) carried out annually from 2001 to 2008 were obtained from NACO [8,9]. BSS data was used to find out the behavioral trends, and also to estimate condom requirement at state level. Mapping studies conducted by NACO and other agencies were used for estimation of size of the high risk group (HRG) populations. District-level mapping estimates for HRG populations were computed from NACO supported size estimation surveys in 11 states under NACP III. Mapping surveys conducted by other agencies such as Avahan and AIDS Prevention and Control (APAC) Project etc. were used in rest of the states. In case more than one mapping estimate was available for a district, the higher estimate was considered for the analysis. For districts with no estimate of HRG population, state-specific number of high risk persons per 1000 population was used to estimate the number of high risk persons in the district. District-wise population for 2008 was obtained from the Population Projections of India [10].

The data for this study was provided by National AIDS Control Organization; hence, any requests for provision of data are to be made to National AIDS Control Organization, New Delhi (India).

### Data analyses

District-wise data on condom supplied (free and through social marketing) by targeted intervention (TI) projects was obtained from the records/reports available with the SACS and their Development Partners. In case of missing data following imputation methods were used in order of preference. Missing data in a TI for a particular year was imputed by (i) average condom distribution per high risk person in the same TI during the preceding and succeeding year, missing data for more than one consecutive year was imputed by (ii) the average number of condoms distributed by rest of the TIs in the same district in the corresponding years, missing data for all TIs of a district in a given year was imputed by (iii) 'per high-risk population condom distribution' of the same district for the preceding and succeeding year multiplied by the number of high risk population covered by TI projects in that year in the district, and missing data of all TIs in a district for more than one consecutive year was imputed by (iv) state average for distribution of condoms per high risk group multiplied by the number of high risk group served under TI projects in the district in those years. Most of the missing data (22%) was imputed using first method, some missing data (4%) was imputed using second method and a few missing data (2%) were imputed using third or fourth method.

Condom requirement in each district was calculated by multiplying the estimated number of FSWs in the district (from mapping estimates) with the average annual number of commercial sex acts per FSW reported in the behavioral surveys. The difference in condom requirement and the number of condoms supplied through targeted intervention (TI) projects was termed as 'condom gap'. The condom gap was used as an indicator of TI intervention intensity at district level. Smaller condom gap indicated higher TI intensity. Districts were ranked state-wise into TI intensity quartiles by using median condom gap from 1995 to 2008.

HIV sentinel surveillance (HSS) data was used to compute district-wise syphilis and HIV prevalence trend among FSWs and young pregnant women (15-24 year olds) in each state. As these trends were in the same direction, combined analysis for all the states was carried out.

Odds ratio for HIV prevalence in young antenatal women (15-24 years) were computed in each quartile of the TI intensity districts for each successive year, taking 2001 as reference year, using generalized linear model with proportion of HIV positive women as the dependent variable with logit link and binomial distribution after adjusting for age, education, and place of residence (rural or urban). Robust standard errors were calculated after considering clustering effects due to site. Data was analyzed using the Stata10 software. 95% confidence interval (CI) and p value for trend were used to summarize the results.

## Results

### TI implementation

**Figure 1:**
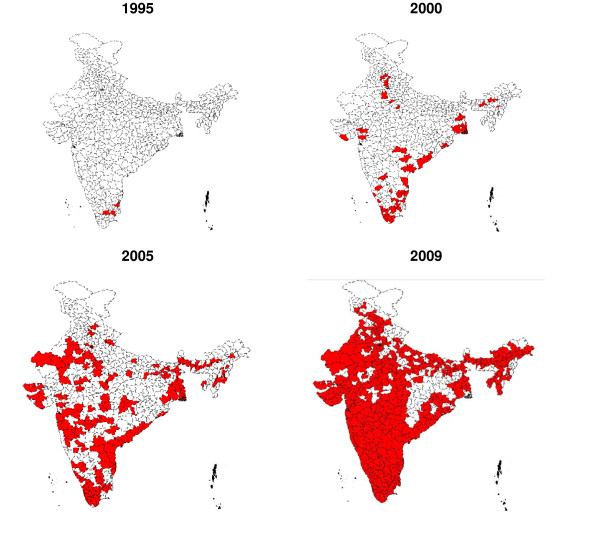
**Geographical distribution of targeted interventions (TIs) for female sex workers (FSWs) in India**.

In the four high HIV prevalence southern states, the number of Targeted Intervention (TI) Projects for FSWs increased from 5 in 1995 to 310 in 2008. In 1995, these projects existed only in Tamil Nadu. By year 2000, composite projects that catered to FSWs covered many districts in the southern states of India. TI projects that catered exclusively to FSWs increased during 2000s covering substantial number of districts, and by 2008 all districts in southern states had been covered by FSW TIs (Figure 1). The number of condoms supplied by targeted intervention (TI) projects also steadily increased from 1995 to 2008.

### Behavioral change

**Figure 2:**
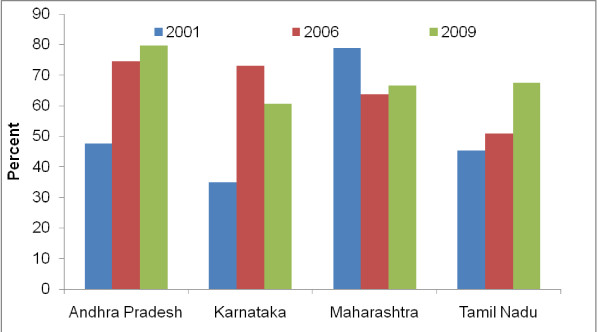
**Men in reproductive age reporting condom use during last sex act with a non-regular sex partner**.

**Figure 3:**
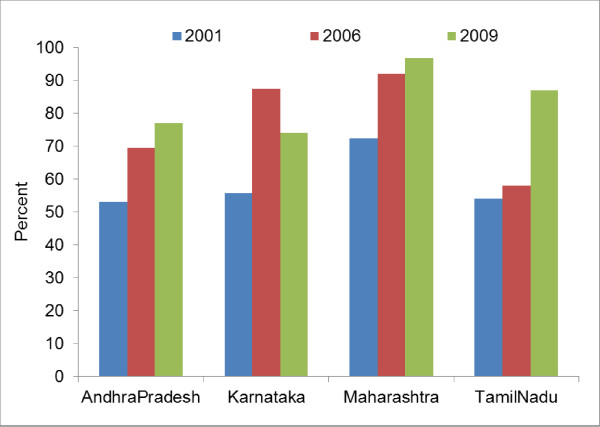
**Female sex workers reporting consistent condom use in the last 30 days during commercial sex acts**.

Figure 2 and 3 show rise in condom use between 2001 and 2009 in the southern states (Andhra Pradesh, Karnataka and Tamil Nadu and Maharashtra). Overall, among men of reproductive age, the condom use during sex with non-regular partner (any sex partner other than spouse for currently married respondents and any sexual partner with whom the respondent does not have sexual intercourse regularly for unmarried or ever married but currently not married men) has increases from 51.7% to 68.6% (p < 0.001). Among FSWs, consistent condom use with last paying clients also increased from 58.6% to 83.7% (p < 0.001).

### Syphilis and HIV trends

Syphilis and HIV prevalence has declined among FSWs in most of the high HIV prevalence states except Tamil Nadu where the prevalence continued to be on the lower side from 2004 to 2007 (Table 1). Before year 2004, sentinel surveillance among FSWs was carried out only in Maharashtra state which had reported very high prevalence of both syphilis and HIV (Table 1).

**Table 1:** *Adjusted Prevalence Trends for HIV and Syphilis in Selected States of India.

Years	2001	2002	2003	2004	2005	2006	2007	2008	P value
**Syphilis: Female Sex Workers (%)**									
Andhra Pradesh	-	-	-	8.0	5.4	2.9	3.9	-	0.1
Karnataka	-	-	-	17.6	4.4	4.2	0	-	< 0.001
Maharashtra	-	39.5	21.1	23.4	13.1	4.5	7.7	-	0.03
Tamil Nadu	-	-	-	2.8	2.5	2.4	1.9	-	0.7
**HIV: Female Sex Workers (%)**									
Andhra Pradesh	-	-	-	17.0	13.0	7.3	9.7	-	0.05
Karnataka	-	-	-	21.6	18.4	8.6	5.3	-	< 0.001
Maharashtra	52.3	54.5	54.3	41.7	23.6	19.6	17.9	-	0.001
Tamil Nadu	-	-	-	4.0	5.5	4.6	4.7	-	0.4
Syphilis: Antenatal Women** (%)									
Andhra Pradesh	-	0.8	2.3	1.9	1.6	1.1	1.0	0.5	0.3
Karnataka	-	0.3	1.7	1.2	1.0	0.4	0.1	0.1	0.2
Maharashtra	-	0.7	0.9	0.5	0.5	0.3	0.2	0.1	< 0.001
Tamil Nadu	-	0.9	0.6	0.3	0.5	0.4	0.4	0.2	< 0.001
HIV: Antenatal Women** (%)									
Andhra Pradesh	1.9	1.7	1.3	1.5	1.6	1.3	1.0	1.2	0.05
Karnataka	1.5	1.7	1.4	1.4	1.4	1.0	0.8	0.8	0.01
Maharashtra	1.7	1.6	1.1	0.9	0.9	0.8	0.7	0.5	< 0.001
Tamil Nadu	1.1	1.1	0.7	0.6	0.5	0.5	0.6	0.4	0.01

*Estimated by generalized linear model with logit link and binomial distribution after adjusting for age, education, and place of residence (rural or urban). Robust standard errors were calculated after considering clustering effects. ** Age: 15-24 yea

Among young antenatal women (15-24 years age group), the prevalence of syphilis continues to be low in each of the southern high prevalence state (Table 1). HIV prevalence has declined in all southern states, although the timing and pace of the declines has not been same in these states. Earliest decline was observed in Tamil Nadu.

### Relationship of targeted interventions (TIs) intensity with HIV prevalence

Intensity of TIs implementation was categorized into quartiles on the basis of the condoms supplied to high risk groups. The requirement of condoms met through TIs was significantly more in high TI intensity quartile district than in the low TI intensity quartile districts. In the high TI program intensity districts, on an average, 28.8% of the condom demand per year was met through TIs (186 condoms/FSW/year), while only 6.3% of the condom demand was met (45 condoms/FSW/year) through TIs in the low TI intensity districts.

**Figure 4:**
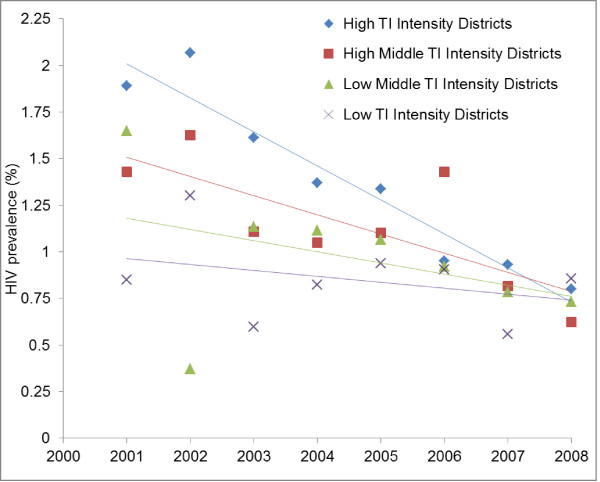
**HIV prevalence trends among young antenatal clinic attendees (15-24 years) according to the intensity of targeted interventions (TI) implementation in Southern states of India**.

In the high TI intensity quartile districts, HIV prevalence in young antenatal women (15-24 years) declined from 1.9% in 2001 to 0.8% in 2008 (p < 0.001), but the low TI intensity districts did not show any significant decline during this period (0.9% in 2001 as well in 2008, p > 0.05). HIV prevalence trend among young antenatal women are presented in the high (Q1), high middle (Q2), low middle (Q3) and low (Q4) TI intensity quartile districts in Figure 4.

Additional File 1: Table S1 presents HIV prevalence odds ratio from reference year 2001 to 2008 in the four TI intensity quartile district after adjusting for age, education, area of residence (urban or rural). Compared to reference year 2001, odd ratio of being HIV positive among young (15-24 years) antenatal women declined to 0.42 (95% CI 0.28, 0.62) in 2008 in the high TI intensity districts whereas there was no significant decline in the low TI intensity districts (OR: 1.01; 95% CI 0.67, 1.50).

## Discussion

In India, focused interventions started well before 1995 in the Metropolitan cities like Chennai, Mumbai and Kolkata, the epicenters of HIV epidemic [11-13]. Since then, targeted interventions (TIs) have been standardized and consistently scaled up. A gradual increase in TIs, especially among FSWs has been seen in southern states of India (Figure 1). Support for these targeted interventions is derived from the 'standard core group theory' based on sexual networks in African and Western societies [14]. Predictions from mathematical models also suggest that a package of interventions focusing on condom use in commercial sex work and treatment of STIs would be sufficient to curtail and ultimately virtually eliminate the HIV epidemic in India [15]. Some observational studies have suggested that intensive implementation of such programmatic interventions results in a more rapid decline in HIV prevalence [11-13,15]. However, rigorous randomized controlled trials have not been conducted to test the 'standard core group theory'. Now, it is ethically not possible to conduct randomized experiments as there is hardly any FSW population without TIs in India.

We have used a semi-experimental design in which HIV prevalence reductions among young pregnant women were compared in large geographical areas (districts) stratified on the basis of the intensity of targeted intervention (TI) implementation as reflected by the program data on condom distribution. Several confounding variables such as age, education, residence etc. were adjusted in the analysis. A statistically significant steeper decline of HIV prevalence in young antenatal women observed in the high intensity targeted intervention districts seems to indicate that the package of targeted interventions have played an important role in bringing about such a decline (Figure 4). However, these results should be interpreted with caution as program data on condom distribution was collected retrospectively from the records. Some missing values had to be imputed. Though the imputation method employed in the study was such that it has only a minimal effect on the inter-district variability. Moreover, sensitivity analysis done by varying the imputed value by 20% on either side, did not affect the overall categorization of the districts. Even without imputation, comparison of high versus low TI intensity districts shows similar results but at slightly lower magnitude. Therefore, we consider that the comparison of trends in the high and low TI intensity districts (extreme quartiles) is valid. Validity of the reported program data on condom distribution by TIs was checked by comparing it with the sources of condoms (purchased from private sources or obtained from targeted intervention project) reported in the behavior surveys. These behavioral surveys have been conducted in various population groups by different agencies at different times using a standard methodology in representative samples and are very likely reflective of actual behavior trends, as is also supported by reviewers of their methodologies [16-18].

Lack of sentinel surveillance data from sufficiently large numbers of FSWs prior to 2004, especially in the low TI intensity districts, was a limitation, hence, temporal relationship of the trends among FSWs and pregnant women could not be established. Behavior surveillance surveys have provided aggregated data at the state level; hence, analysis of primary data that could have adjusted for confounding could not be done for behavioral trends. Sample size was also not sufficient to compute safe sex behavior trends at district level. Hence, a causal link between the TI intensity, behavior change among FSWs and general population males and changes in STI and HIV among young FSWs and antenatal women could not be established directly in the district level analysis. However, state level analysis does indicate existence of a result chain from rise in program effort for targeted intervention (TI) implementation (Figure 1), increase in consistent condom use among FSWs and in multi partner sex among general population males (Figure 2 &3), to decline in the prevalence of syphilis and HIV among FSWs and young antenatal women (Table 1). Studies conducted in India and other countries, among FSWs and other high risk populations, have also reported increase in condom use following focused intervention programs [6,11,13,19-23], and decline in HIV prevalence have also been observed earlier in Sonagachi [5] and Mysore [24] in India and in Thailand [25].

Beside program interventions, other factors such as mobility and mortality can also contribute to the decline in HIV prevalence. Though, decline of HIV prevalence among young antenatal women is more likely due to the reduction in new infections. Mathematical models have also predicted impact of targeted interventions on HIV transmission, provided these interventions achieve large reductions in unprotected FSW-client sex [15,26]. A recent study using reported condom use rates in commercial sex, has estimated HIV decline in India of similar magnitude as is reported in this study [27]. These predictions are consistent with the pivotal role of sex workers in HIV transmission in India. FSWs act as non-regular sex partners and have high HIV prevalence. Though commercial sex acts constitute only a small proportion of the total sexual acts in India, these form the main portal of heterosexually transmitted HIV.

Even though there is scant evidence of HIV trends before 2000 in India, arguments for natural decline in HIV prevalence appear implausible. Natural evolution of HIV infection cannot explain the very large annual decline in HIV infections and coincident declines in syphilis prevalence seen after 2000. There have been no well-described changes in the fitness of the HIV virus, or changes in the prevalence of host-immunity. There have been arguments, however, that the observed declines in HIV prevalence among antenatal women in Avahan covered districts in Karnataka state are unlikely to be explained by targeted interventions alone [28]. Other factors, like exhaustion of susceptible pool or high mortality may have contributed in the decline in HIV prevalence. A more plausible explanation of declining HIV prevalence in young pregnant women in India is the initiation of focused public health programs in late 1980s and early 1990s in the epicenters of the epidemic which were later expanded to large scale targeted interventions to bring about behavior change. It seems that large scale up of TIs that focused on the high risk groups especially in a concentrated epidemic setting have played a key role in curtailing the heterosexually driven epidemic in India.

## Conclusions

HIV epidemic in India has remained more contained than was initially predicted. It appears that increased condom use between sex workers and their clients that took place as a result of preventive interventions; especially the targeted interventions seem to have contributed to the decline in HIV prevalence among young pregnant women.

## Abbreviations used in the paper

ANC: Antenatal Clinic; BSS: Behavioral Surveillance Survey; CMIS: Computerized Management Information System; CI: Confidence Interval; FSW: Female Sex Workers; HRG: High Risk Group; HSS: HIV Sentinel Surveillance; IDU: Injection Drug Users; NACO: National AIDS Control Organization; NACP: National AIDS Control Program; MSM: Men who have Sex with Men; SACS: State AIDS Control Society; STI: Sexually Transmitted Infections; TI: Targeted Interventions

## Conflict of interests

The authors declare that they have no competing interests.

## Authors' contributions

RK, SMM, SPanda and SV conceived the study. PVM, MK, SVG, TS, AKS, AR, RS and BM prepared the study design and data collection instruments. AKS, SP, TS, SS, TR, VT and NDP supervised data collection and did quality checks. SPrinja, PB and NKV analyzed program service data and HIV sentinel surveillance data. TS interpreted the analysed data and wrote the first draft of manuscript. RK, SMM, SPanda, PVM, MK, TS and SPrinja provided critical inputs to data interpretation and revision of the manuscript. All authors have read and finally approve the version submitted to the journal for publication.

## Pre-publication history

The pre-publication history for this paper can be accessed here:

http://www.biomedcentral.com/1471-2458/11/549/prepub

## Supplementary Material

Additional File 1 **Table S1**. HIV prevalence odds ratio (OR) trends among young antenatal clinic attendees (15-24 years) according the intensity of targeted intervention (TI) implementation in selected Indian states. Click here for file
