# Supplementary material for: Impact of targeted interventions on heterosexual transmission of HIV in India
Source: BMC Public Health. 2011 Jul 11;11:549. doi: 10.1186/1471-2458-11-549 (PMC3152907; doi:10.1186/1471-2458-11-549)
Supplement: Additional File 1 — Table S1. HIV prevalence odds ratio (OR) trends among young antenatal clinic attendees (15-24 years) according the intensity of targeted intervention (TI) implementation in selected Indian states. [file 1471-2458-11-549-S1.DOC]

**Table S1: HIV prevalence odds ratio (OR) trends among young antenatal clinic attendees (15-24 years) according the intensity of targeted intervention (TI) implementation in selected Indian states.**

|  |  | **High**  **TI intensity**  **Districts** |  |  | **High Middle TI intensity districts** |  |  | **Low Middle TI intensity districts** |  |  | **Low**  **TI intensity districts** |  |
| --- | --- | --- | --- | --- | --- | --- | --- | --- | --- | --- | --- | --- |
| **Year** | **OR** | **Lower**  **95% CI** | **Upper**  **95% CI** | **OR** | **Lower**  **95% CI** | **Upper**  **95% CI** | **OR** | **Lower**  **95% CI** | **Upper**  **95% CI** | **OR** | **Lower**  **95% CI** | **Upper**  **95% CI** |
| **2001*** |  |  |  |  |  |  |  |  |  |  |  |  |
| **2002** | 1.09 | 0.76 | 1.58 | 1.14 | 0.59 | 2.2 | 0.22 | 0.09 | 0.57 | 1.53 | 1.21 | 1.95 |
| **2003** | 0.85 | 0.59 | 1.23 | 0.77 | 0.51 | 1.17 | 0.68 | 0.35 | 1.32 | 0.7 | 0.48 | 1.03 |
| **2004** | 0.72 | 0.49 | 1.05 | 0.73 | 0.49 | 1.09 | 0.67 | 0.36 | 1.27 | 0.97 | 0.66 | 1.4 |
| **2005** | 0.7 | 0.48 | 1.03 | 0.77 | 0.49 | 1.2 | 0.64 | 0.34 | 1.23 | 1.11 | 0.74 | 1.66 |
| **2006** | 0.49 | 0.34 | 0.73 | 0.62 | 0.42 | 0.91 | 0.55 | 0.29 | 1.06 | 1.07 | 0.69 | 1.64 |
| **2007** | 0.49 | 0.33 | 0.72 | 0.57 | 0.37 | 0.86 | 0.47 | 0.24 | 0.97 | 0.65 | 0.42 | 1.01 |
| **2008** | 0.42 | 0.28 | 0.62 | 0.43 | 0.28 | 0.67 | 0.44 | 0.23 | 0.84 | 1.01 | 0.67 | 1.5 |

* Reference Year
